# MCP/CCR2 Signaling Is Essential for Recruitment of Mesenchymal Progenitor Cells during the Early Phase of Fracture Healing

**DOI:** 10.1371/journal.pone.0104954

**Published:** 2014-08-18

**Authors:** Masahiro Ishikawa, Hiromu Ito, Toshiyuki Kitaori, Koichi Murata, Hideyuki Shibuya, Moritoshi Furu, Hiroyuki Yoshitomi, Takayuki Fujii, Koji Yamamoto, Shuichi Matsuda

**Affiliations:** 1 Department of Orthopaedic Surgery, Kyoto University Graduate School of Medicine, Kyoto, Japan; 2 Department of the Control for Rheumatic Disease, Kyoto University Graduate School of Medicine, Kyoto, Japan; 3 The Center for Innovation in Immunoregulative Technology and Therapeutics, Kyoto University Graduate School of Medicine, Kyoto, Japan; 4 Center for the Promotion of Interdisciplinary Education and Research, Kyoto University, Kyoto, Japan; University-Hospital of Parma, Italy

## Abstract

**Objective:**

The purpose of this study was to investigate chemokine profiles and their functional roles in the early phase of fracture healing in mouse models.

**Methods:**

The expression profiles of chemokines were examined during fracture healing in wild-type (WT) mice using a polymerase chain reaction array and histological staining. The functional effect of monocyte chemotactic protein-1 (MCP-1) on primary mouse bone marrow stromal cells (mBMSCs) was evaluated using an *in vitro* migration assay. MCP-1^−/−^ and C-C chemokine receptor 2 (CCR2)^−/−^ mice were fractured and evaluated by histological staining and micro-computed tomography (micro-CT). RS102895, an antagonist of CCR2, was continuously administered in WT mice before or after rib fracture and evaluated by histological staining and micro-CT. Bone graft exchange models were created in WT and MCP-1^−/−^ mice and were evaluated by histological staining and micro-CT.

**Results:**

*MCP-1* and *MCP-3* expression in the early phase of fracture healing were up-regulated, and high levels of MCP-1 and MCP-3 protein expression observed in the periosteum and endosteum in the same period. MCP-1, but not MCP-3, increased migration of mBMSCs in a dose-dependent manner. Fracture healing in MCP-1^−/−^ and CCR2^−/−^ mice was delayed compared with WT mice on day 21. Administration of RS102895 in the early, but not in the late phase, caused delayed fracture healing. Transplantation of WT-derived graft into host MCP-1^−/−^ mice significantly increased new bone formation in the bone graft exchange models. Furthermore, marked induction of MCP-1 expression in the periosteum and endosteum was observed around the WT-derived graft in the host MCP-1^−/−^ mouse. Conversely, transplantation of MCP-1^−/−^ mouse-derived grafts into host WT mice markedly decreased new bone formation.

**Conclusions:**

MCP-1/CCR2 signaling in the periosteum and endosteum is essential for the recruitment of mesenchymal progenitor cells in the early phase of fracture healing.

## Introduction

The prevalence of osteoporosis is increasing with the aging of society. In particular, osteoporotic fractures are a major public health problem with a low one-year patient's survival rate [1], [2]. Management of the fracture is difficult because of poor bone quality, and there is a high risk of fixation failure and nonunion. To avoid these difficulties, numerous attempts have been made to develop techniques to improve fracture healing, including addition or injection of bone-forming factors or cells such as mesenchymal stem/progenitor cells [3], [4]. However, treatment options remain below expectations despite vigorous attempts to find new useful therapies. One major reason is that the mechanisms responsible for fracture healing are complex and not fully understood. Hence, elucidating the mechanisms involved in fracture healing is fundamental to developing novel therapeutic strategies to improve fracture healing.

Normal fracture healing follows a unique, distinct healing process, which can be divided into three overlapping phases: inflammation, repair and remodeling [5]. Among these three phases, the repair and remodeling phases largely recapitulate the process of normal bone development [6]. In contrast, the inflammation phase is a unique process that is not observed in the organogenesis of bone and develops after birth to induce bone repair. Previous studies have shown that inflammation plays a pivotal role in fracture healing [7], [8] and that mesenchymal stem/progenitor cells are systemically or locally recruited to the fracture site in the early inflammatory phase [5], [9]. Many proinflammatory cytokines and chemokines are released from the fracture site in the early inflammatory phase [9], [10]. Chemokines are small, chemoattractant cytokines that play key roles in the recruitment of leukocytes to sites of inflammation and injury. Studies have shown that stem/progenitor cell migration and organ-specific recruitment are regulated by chemokines and their receptors [11]–[13]. In addition, mesenchymal stem/progenitor cells express a variety of chemokine receptors [14], and chemokine-mediated mesenchymal stem/progenitor cell migration has been shown *in vitro* and *in vivo* [15], [16].

Over the past decade, attention has focused on stem/progenitor cells because of their pivotal role in tissue regeneration. Mesenchymal stem/progenitor cells exhibit extensive tropism for tissue injury sites [17]. These cells differentiate into mesenchymal lineage cells when exposed to appropriate environmental cues and can promote tissue repair of many organs, including bone. In addition, mesenchymal stem/progenitor cells appear to exist in almost all tissues, including bone marrow, muscle and the periosteum, and if not present, can reach tissues via the blood circulation [18]. Therefore, mesenchymal stem/progenitor cells can be recruited from the circulation or surrounding tissues and participate in the repair of the injured organs [12], [19].

Several studies have shown that systemically infused mesenchymal stem/progenitor cells can migrate to, and participate in, the repair of injured tissue [20]–[22]. We have previously demonstrated in a bone graft model that stromal cell-derived factor-1 (SDF-1) is induced in the periosteum of fracture sites and promotes endochondral bone repair by recruiting C-X-C chemokine receptor 4 (CXCR4)-expressing mesenchymal stem/progenitor cells [23]. Thus, mesenchymal stem/progenitor cell therapy may be a novel therapeutic strategy to improve fracture healing. To develop an efficient therapy, it is crucial to elucidate the precise mechanisms for recruitment of mesenchymal stem/progenitor cells to the fracture site. However, these mechanisms, especially during the early inflammatory phase, are largely unknown.

To identify the factor(s) essential for normal fracture healing, we used a polymerase chain reaction (PCR) array and mouse rib fracture model in which cell potential is non-impaired by surgical intramedullary fixation. We also used an exchange-graft model to show gain- or loss-of-function. We demonstrate herein that the expression level of monocyte chemotactic protein-1 (MCP-1) is up-regulated exclusively in the early fracture phase and that MCP-1 is expressed at the periosteum and endosteum of the fractured bones. Gain- and loss-of-function studies showed that the MCP-1/C-C chemokine receptor 2 (CCR2) axis is crucial in the early phase of fracture healing. In summary, these results indicate that the MCP-1/CCR2 axis provides essential signaling for normal bone healing and may be a novel, potent therapeutic target for fracture healing.

## Materials and Methods

### Reagents

Recombinant mouse MCP-1 and MCP-3 were purchased from Abcam (Cambridge, MA, USA). CCR2 antagonist (RS102895) was purchased from Sigma (St. Luis, MO, USA).

### Mouse rib fracture model

All animal studies were conducted in accordance with principles and procedures approved by the Kyoto University Committee of Animal Resources. Surgeries were undergone under anesthesia with diethylether, and mice were euthanatized with cervical dislocation upon sacrifice. Mouse rib fracture models were created using 6-week-old C57BL/6 wild-type (WT), MCP-1^−/−^ and CCR2^−/−^ mice, as described previously [24]. Five mice from each fracture group were sacrificed 0, 1, 2, 3, 5, 7, 10, 14, 21, and 25 days after fracture. To evaluate the inhibitory effect of the receptor antagonist, the mice received continuous administration of the selective CCR2 antagonist, RS102895. RS102895 was dissolved in dimethyl sulfoxide (DMSO) and via an osmotic pump (model 1002; Durect, Cupertino, CA, USA), was delivered to a total of 10 mg/kg/day, beginning 2 days before or 4 days after rib fracture, and until day 12. In the control group, DMSO alone was administered for 14 days.

### Femoral segmental bone graft transplantation model

A mouse segmental bone graft model was created using 6-week-old C57BL/6 WT and MCP-1^−/−^ mice as described previously [25]. Briefly, 4-mm of mid-diaphyseal segmental bone was removed from the femur of the donor mouse. The graft was dissected carefully to remove the muscle and bone marrow without compromising the periosteum, and segmental bone derived from a WT or MCP-1^−/−^ mouse was transplanted immediately into a 4-mm segmental defect in a host WT or MCP-1^−/−^ mouse. Four groups of segmental bone graft models were used: MCP-1^−/−^ donor to MCP-1^−/−^ host [knockout (KO)-to-KO], WT donor to MCP-1^−/−^ host (WT-to-KO), WT donor to WT host (WT-to-WT), and MCP-1^−/−^ donor to WT host (KO-to-WT). The bone graft was stabilized using a 25 G stainless pin placed through the intramedullary marrow cavity, and the mice were sacrificed on day 21 after the surgery for RNA extraction and histological analysis.

### Micro-CT analysis

Mice were sacrificed postoperatively for micro-computed tomography (micro-CT) imaging on days 7 (femoral bone graft model) and 21 (rib fracture model). The rib and femur were scanned using a micro-CT system (SMX-100CT-SV3; Shimadzu, Tokyo, Japan) at 2400 views, five frames per view, 40 kV, and 40 µA. Three-dimensional (3D) images were rendered and evaluated using VG Studio MAX (Nihon Visual Science Software, Tokyo, Japan). The newly formed callus was spatially segmented from the native cortical bone in the two-dimensional (2D) tomograms, the 3D images of the callus were rendered, and the total volume was measured on the digitally extracted callus tissue. The newly formed calluses in a region of interest covering the entire length of the bone graft, including 1 mm of the host bone at both proximal and distal bone graft junctions, were analyzed to determine bone graft healing.

### RNA extraction, quantitative real-time PCR and PCR array

Total RNA was extracted from mouse rib and femoral bone graft specimens as described previously [26]. A PCR array was performed to measure mRNA levels for chemokines during the fracture healing process. Two micrograms of RNA was processed using an RT2 First Strand Kit (SA Biosciences, Frederick, MD, USA) according to the manufacturer's specifications. Quantitative PCR analysis for chemokines and receptors was assessed using a chemokine array (Chemokines & Receptors PCR Array, Mouse, PAMM-022, SA Biosciences). We analyzed the data using the RT2 profiler PCR Array Data Analysis software (SA Biosciences). The change in gene expression level determined by PCR array analysis was confirmed by quantitative real-time PCR. All gene expression data were normalized against glyceraldehyde phosphate dehydrogenase (GAPDH).

### Histological analysis

Rib and femur specimens were processed as paraffin-embedded sections and stained with hematoxylin and eosin and were subjected to immunohistochemical analyses as described previously [23], [24].

### *In vitro* chemotaxis assay

*In vitro* cell migration of primary mouse bone marrow stromal cells (mBMSCs) (1.0×10^5^ cells/100 µl) was assessed using Transwell inserts with an 8-µm pore membrane, as described previously [23]. For the chemotaxis assay, different concentrations of MCP-1 or MCP-3 (0, 10, or 100 ng/ml) in 500 µl of medium were applied to the lower chambers. For the inhibition assay, RS102895 (400 nM) was also applied to the lower chambers. After 24 h of incubation, the migrated cells were counted under light microscopy.

### Primary cell culture, cell line, osteogenesis and chondrogenesis assay

For the osteogenesis assay, mBMSCs were harvested and cultured as described previously [23]. mBMSCs were cultured with osteogenic base media (R&D Systems, Minneapolis, MN, USA) for 2 days. On reaching 70% confluence, the medium was replaced with osteogenic differentiation medium and was changed every 3 days thereafter. In some experiments, recombinant mouse MCP-1 (200 ng/ml) was added every 3 days with each medium change. On day 14 after plating, cells were harvested for alizarin red staining and gene expression analysis. For the chondrogenesis assay, ATDC5 cells were cultured and maintained in Dulbecco's Modified Eagles Medium (DMEM) and Ham's F-12 at a 1∶1 ratio with 5% fetal bovine serum (FBS) supplemented with insulin (10 mg/mL, Sigma), transferrin (5.5 mg/mL, Sigma), and sodium selenite (5 ng/mL, Sigma) to induce chondrocyte differentiation as described previously [27]. The medium was changed every 2 days thereafter. In some experiments, MCP-1 (0, 20, 100 or 200 ng/ml) was simultaneously added every 2 days with the medium change. On day 28 after plating, cells were harvested for gene expression analysis.

### Statistical analysis

Data are presented as means ± standard error of the mean (SEM). We analyzed the data using GraphPad Prism Version 5.00 (GraphPad software). Statistical comparisons between two groups were performed using a Student's two-tailed *t* test. Differences between three groups were analyzed using the Bonferroni method. *P* values <0.05 were considered significant.

## Results

### Chemokine expression profile in the early phase of fracture healing

**Figure 1:**
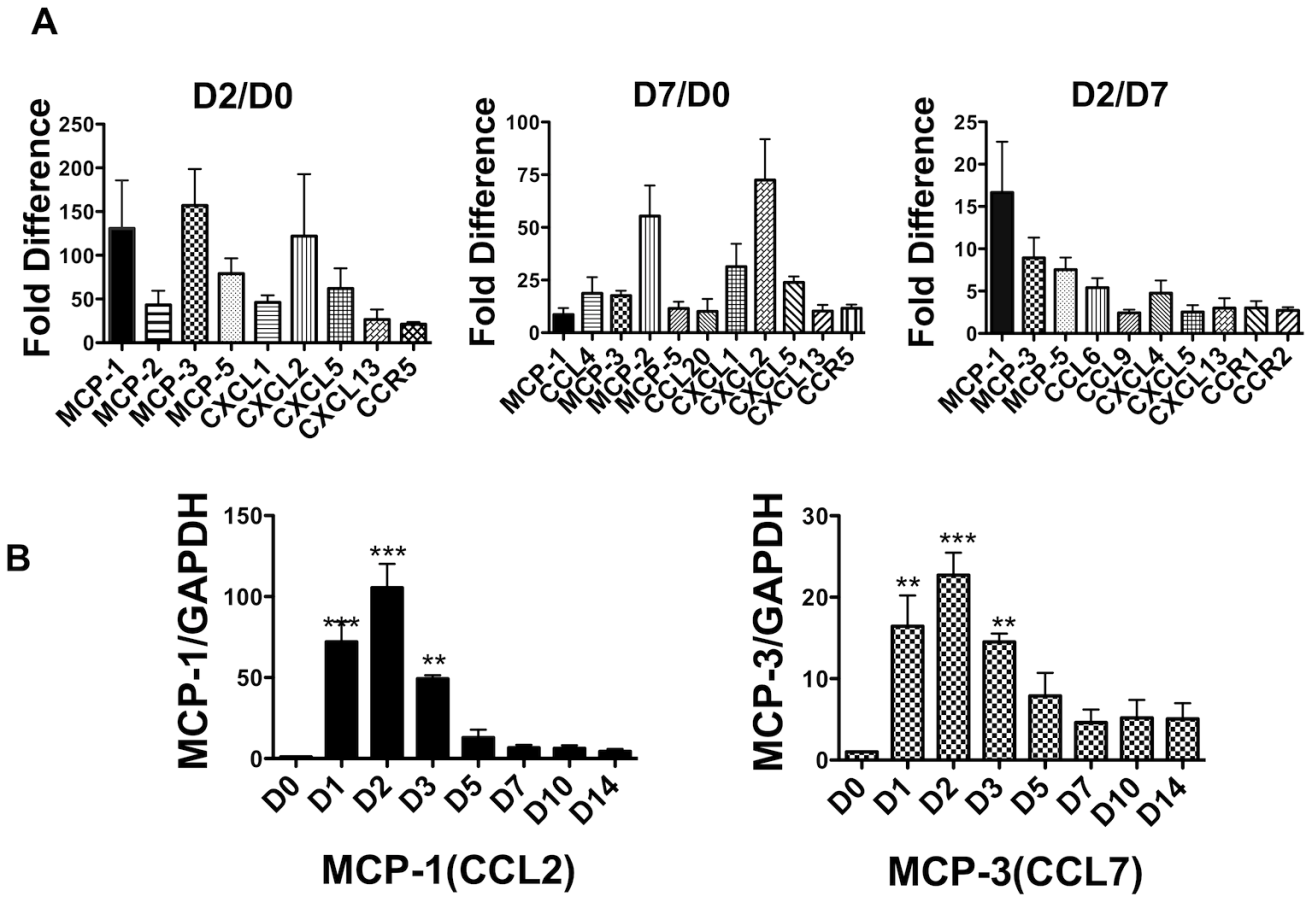
The expression profiles of chemokines and their receptors during fracture healing. Expression levels during fracture healing were examined using a rib fracture model. **A**: PCR array data for up-regulated chemokines and their receptors during fracture healing are shown. Expression levels were compared between days 0 and 2, days 0 and 7, and days 2 and 7. **B**: Time course of *MCP-1* and *MCP-3* mRNA expression in a rib fracture model, as analyzed by real-time PCR. Expression levels are the fold change from day 0 levels. Values are means ± SEM of more than four separate experiments. **P<0.01 ***P<0.001 compared with the day 0 group.

We first used PCR array to investigate the expression profile of chemokines during fracture healing in the WT mouse rib fracture model. The PCR array analysis showed that the expression levels of *MCP-1* and *MCP-3* were significantly higher on day 2 compared with days 0 and 7 (Figure 1A). *MCP-1* and *MCP-3* expression levels were more than 100 times higher on day 2 than on day 0. *MCP-1* and *MCP-3* expression levels on day 2 were five times higher than those on day 7. To confirm the PCR array data, we next examined the gene expression levels for *MCP-1* and *MCP-3* during the fracture healing process. Consistent with the PCR array analysis, the expression of *MCP-1* and *MCP-3* increased during the early phase of fracture healing (Figure 1B). *MCP-1* and *MCP-3* were expressed on day 1 and their expression peaked on day 2. By day 7, expression of both genes had declined markedly.

### *In vivo* expression of MCP-1 during the early inflammatory phase of fracture healing

**Figure 2:**
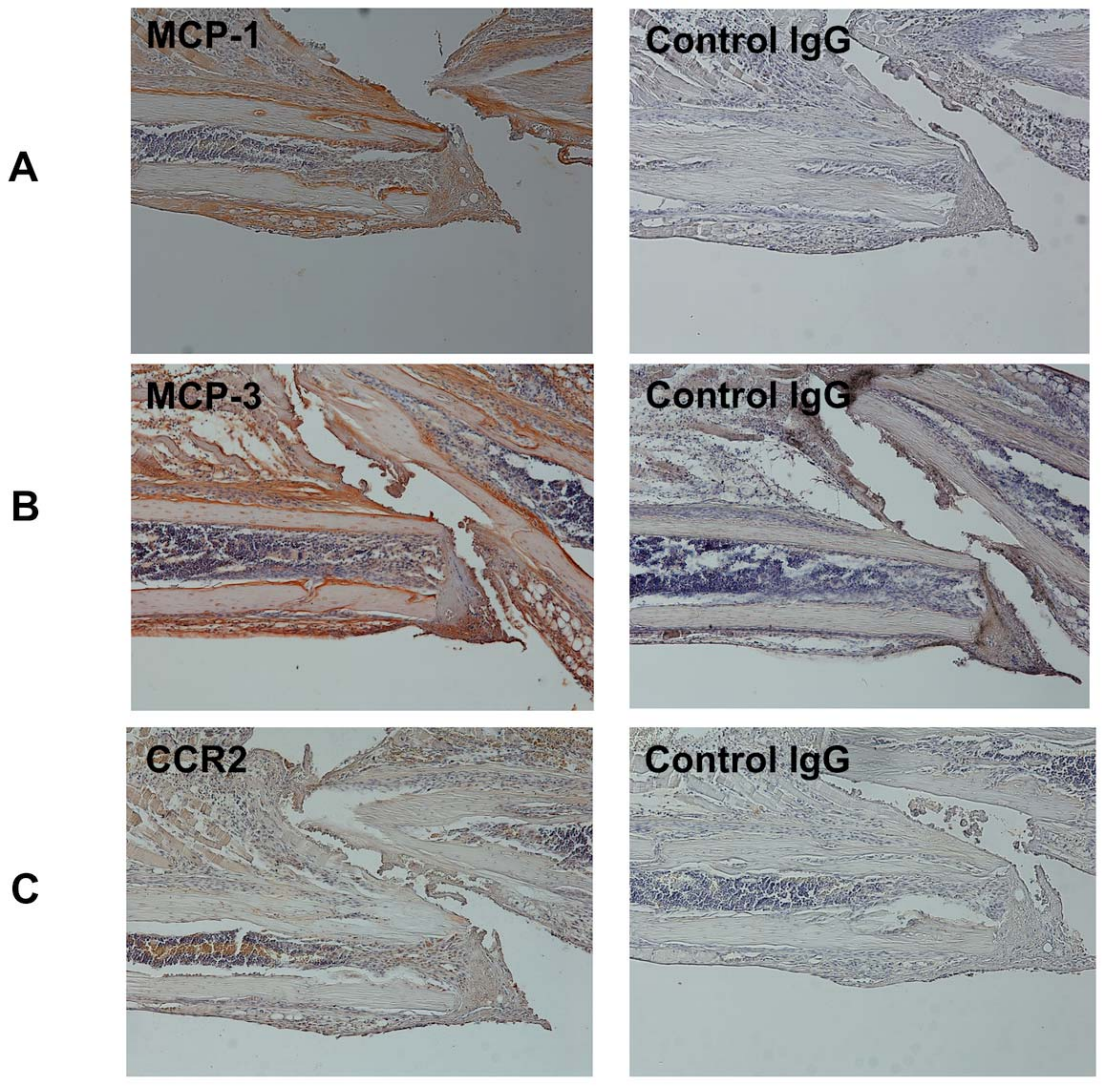
Immunohistochemical analysis of WT rib fracture on day 3. A, B, Protein expression levels of MCP-1 (**A**) and MCP-3 (**B**) were identified at the periosteum and endosteum on day 3. **C**, CCR2-positive cells were predominantly found within the bone marrow and surrounding tissues on day 3. Original magnification, 40×. Middle panel, high-magnification views (original magnification, 200×). The result is representative of three separate experiments.

Because CCR2 is the major and common receptor for MCP-1 and MCP-3, we focused on the expression of MCP-1, MCP-3 and CCR2 in fracture healing, especially during the early inflammatory phase. To confirm the localization of MCP-1, MCP-3 and CCR2 expression during the early inflammatory phase of fracture healing, we used immunohistochemistry to examine rib fracture healing in WT mice. Low expression levels of MCP-1 and MCP-3 were observed at the periosteum in the unfractured rib, (Figures S1A, B) and high levels of MCP-1 and MCP-3 protein were observed at the periosteum and endosteum on day 3 in the fractured rib (Figure 2A, B). Conversely, little or no CCR2 staining was detected in the unfractured rib (Figure S1C), and on day 3, CCR2-positive cells were found predominantly within the bone marrow and surrounding tissues (Figure 2C).

### MCP-1 induces mBMSCs migration *in vitro*

**Figure 3:**
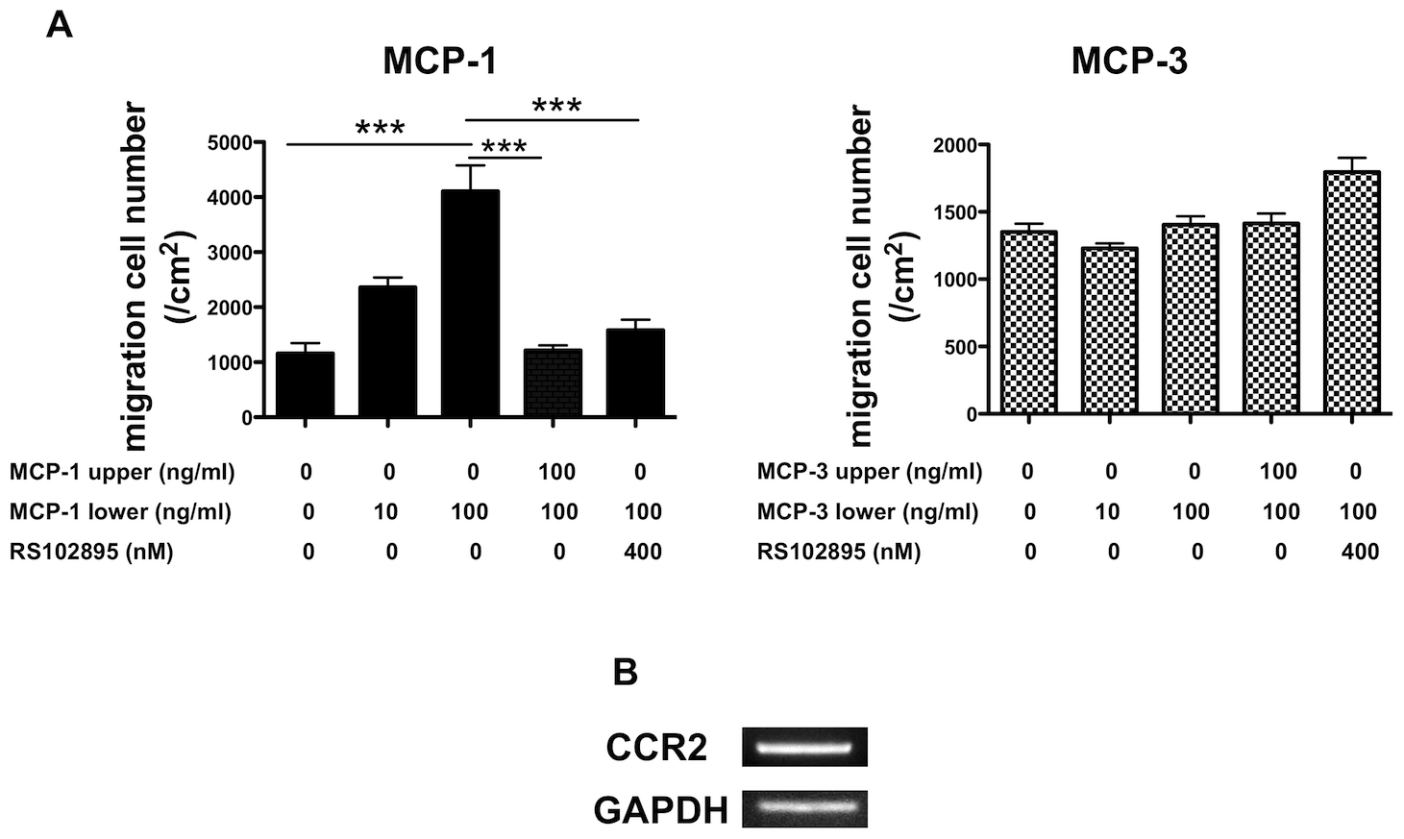
Effect of MCP-1 on cell migration. **A**: *In vitro* migration assay. mBMSCs were stimulated by MCP-1 or MCP-3 at indicated doses and RS102895 at 400 nM. Cells that migrated to the undersurface of the membrane were counted. Numbers of cells are represented as cell number per cm^2^. Values are means ± SEM (*n* = 5, respectively). **B**: Expression of *CCR2* mRNA in mBMSCs. Data are shown as means ± SEM. *P<0.05.

To examine the functional roles of MCP-1 and MCP-3 signaling in fracture healing, we first examined whether MCP-1 and MCP-3 could induce the migration of mBMSCs. MCP-1 significantly increased mBMSC migration in a dose-dependent manner, whereas MCP-3 did not (Figure 3A). Because MCP-1 and MCP-3 act through their receptor CCR2, we examined the expression of CCR2 in these cells. Consistent with a previous report [30], RT-PCR analysis showed that CCR2 was expressed in mBMSCs (Figure 3B). We also examined whether CCR2 mediates MCP-1-induced migration of mBMSCs. RS102895 (400 nM) effectively inhibited the MCP-1-induced migration of mBMSCs (Figure 3A). Because MCP-3 did not affect migration of the cells, we therefore focused on MCP-1. To further investigate possible roles of MCP-1, we next examined osteogenic differentiation of mBMSCs in response to MCP-1. Isolated mBMSCs were capable of spontaneously differentiating into alizarin red-positive cells and showed increased levels of *Runx2*, *osterix* and *alkaline phosphatase (ALP)* in the osteoinduction media on day 14. However, alizarin red staining revealed no difference between osteoinduced mBMSCs with or without MCP-1 (Figure S2A). Similarly, no difference in the gene expression of *Runx2, osterix, or ALP* was observed in cells treated with or without MCP-1 (Figure S2A). Because MCP-1 did not affect the osteogenic differentiation of mBMSCs, we also examined whether MCP-1 affects chondrogenic differentiation using ATDC5 cells *in vitro*. Alcian blue staining showed that the presence of MCP-1 was not associated with any obvious differences in cells treated with or without MCP-1 on day 28. Moreover, *SOX9, Col-2* and *Col-10* showed similar expression patterns in cells treated with or without MCP-1 (Figure S2B).

### *In vivo* roles of MCP-1 and CCR2 during the early inflammatory phase of fracture healing

**Figure 4:**
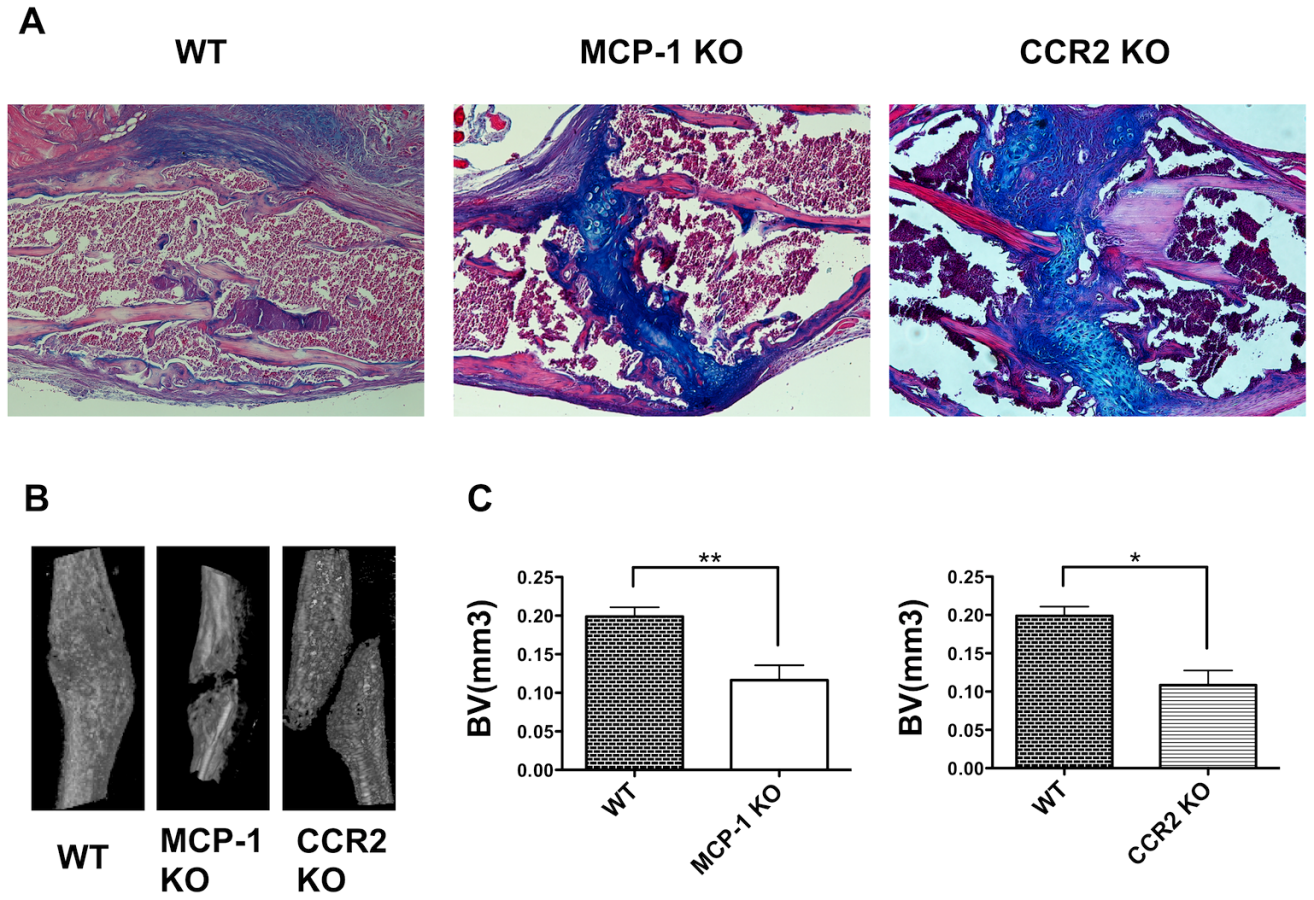
MCP-1^−/−^ and CCR2^−/−^ mice displayed delayed fracture healing *in vivo*. **A**: Histology of the fracture callus stained by hematoxylin-eosin/alcian-blue staining on day 21. **B**: Representative 3D micro-CT image of a fractured rib on day 21. **C**: Newly formed callus volume in the MCP-1^−/−^ and CCR2^−/−^ mice on day 21 was quantified using micro-CT.

To investigate the functional roles of MCP-1 and CCR2 in fracture healing, rib fracture healing was assessed in WT and KO mice. Fracture calluses were examined by micro-CT and histological analysis. Histological analyses showed a smaller proportion of cartilage in the callus in MCP-1^−/−^ mice compared with WT mice on day 7 (Figure S3A). By day 21, fractures had healed in the WT mice and cartilage was almost completely replaced by bone in the callus (Figure 4A). Conversely, the healing processes progressed incompletely in MCP-1^−/−^ and CCR2^−/−^ mice by day 21, and the central area of the cartilaginous callus remained (Figure 4A). By day 25, a bridging callus was apparent in MCP-1^−/−^ and CCR2^−/−^ mice, and was similar in appearance to that observed in WT mice on 21 day (Figure S3B). The callus volume was significantly smaller in both MCP-1^−/−^ and CCR2^−/−^ mice than in WT mice on day 21 (Figure 4B, C).

**Figure 5:**
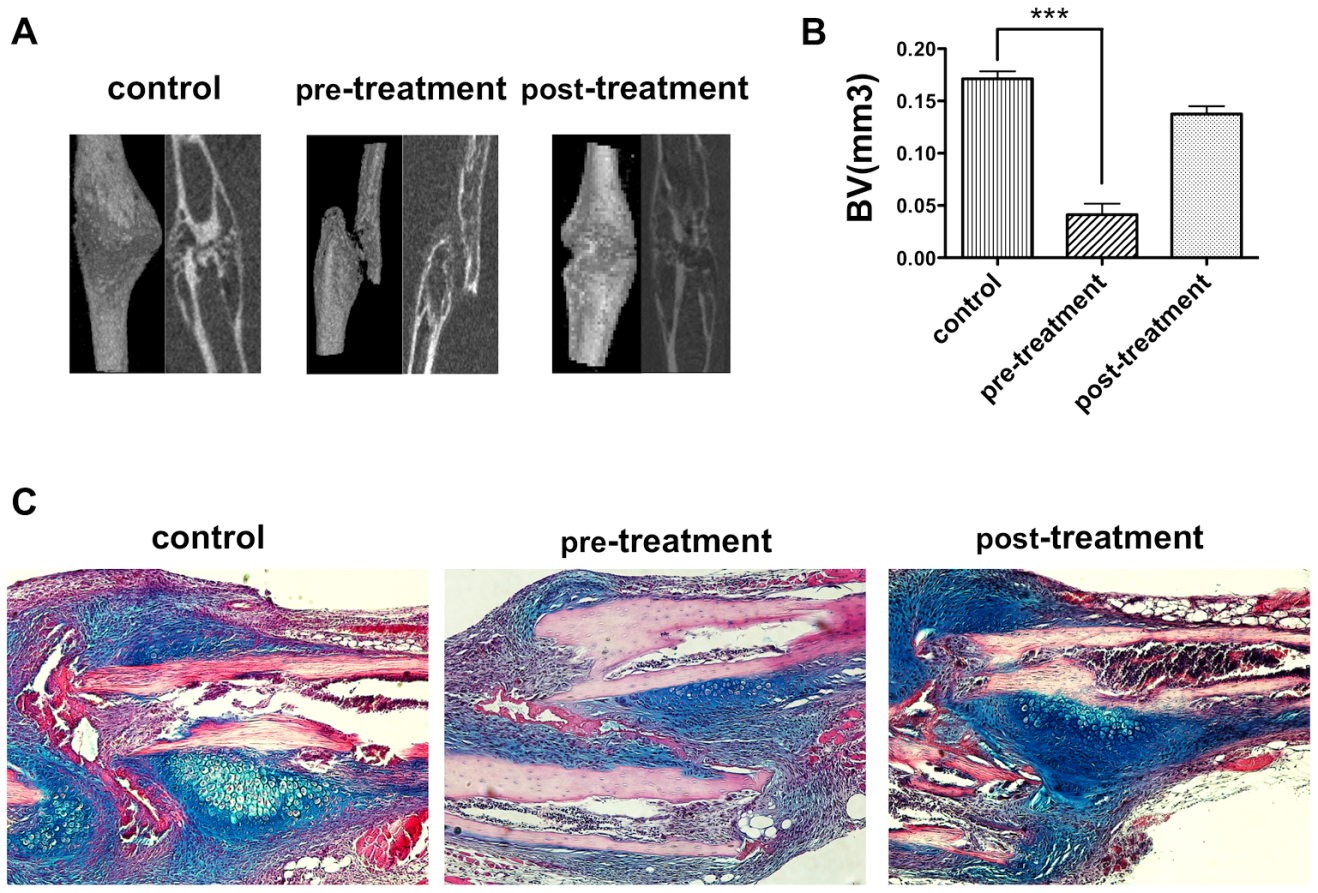
Blockade of CCR2 in the early phase displayed delayed fracture healing *in vivo*. **A**: WT mice received continuous administration of CCR2 antagonist, RS102895, or DMSO (controls) until day 12, beginning 2 days before or 4 days after rib fracture. In the control group, DMSO was administered as a control for 14 days. Representative micro-CT image of a fractured rib on day 21. **B**: Newly formed callus volume on day 21 in the pre-treatment or post-treatment group was quantified using micro-CT. **C**: Histology of the fracture callus stained by hematoxylin-eosin/alcian-blue staining on day 7.

Next, to elucidate whether the MCP-1/CCR2 axis is involved during the early phase of fracture healing, we continuously administered RS102895 before (pre-treatment) or after (post-treatment) rib fracture. Micro-CT analysis showed delayed fracture healing in the pre-treatment group compared with both the control and post-treatment groups. On day 21, the callus volume was significantly smaller in the pre-treatment group than in the control and post-treatment groups (Figures 5A, B). Histological analysis showed that fractures in both the control and post-treatment groups had healed by day 21 and that cartilaginous tissue was absent in the callus. Conversely, less cartilaginous tissue was observed in the callus in the pre-treatment group on day 7 (Figure 5C), and cartilaginous tissue in a central area of the callus was observed on day 21 (Figure S3C). These results indicate that the MCP-1/CCR2 axis is an essential component during the early phase of fracture healing.

### Periosteal bone formation in grafts from WT mice implanted into MCP-1-deficient mice: gain-of-function

**Figure 6:**
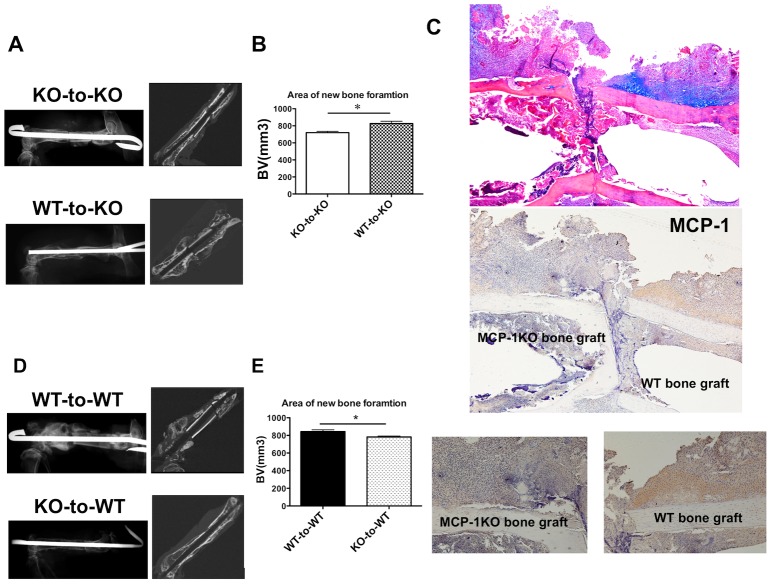
Femoral segmental bone graft exchanging model. Bone exchange surgeries were performed between WT and MCP-1^−/−^ mice as described in the Methods section. Samples were harvested on day 21 for micro-CT and histological analyses. **A and B**, Representative micro-CT images and quantitative analyses demonstrate that WT mouse-derived bone graft caused a significant increase of new bone formation compared with KO mouse-derived bone graft. **C**, Immunohistochemical staining for MCP-1 in MCP-1^−/−^ mice is shown. Pronounced MCP-1 expression at the periosteum and endosteum in the WT graft was observed in the host MCP-1^−/−^ mouse. **D**, Representative micro-CT images and quantitative analyses demonstrate that MCP-1^−/−^ mouse-derived bone graft caused a significant decrease of new bone formation compared with WT mouse-derived bone graft. Values are means ± SEM of more than three separate experiments. *P<0.05.

To examine the roles of MCP-1 at the periosteum and endosteum during fracture healing, we performed gain-of-function studies using a segmental bone graft transplantation model. A segmental bone graft was transplanted from an MCP-1^−/−^ mouse to another MCP-1^−/−^ mouse (KO-to-KO). Micro-CT and histological analysis were used to quantify new bone formation on day 21. Radiologic and micro-CT analyses showed that KO-to-KO transplantation caused a delay in fracture healing on day 21 (Figure 6A). Minimal periosteal bone formation was observed along the surface of the bone graft because of the lack of periosteal bone formation. We next created bone graft exchanging models between MCP-1^−/−^ and WT mice, in which a segmental bone derived from a WT mouse was transplanted into a host MCP-1^−/−^ mouse (WT-to-KO). In contrast to KO-to-KO bone graft transplantation, transplantation of the WT-derived graft into the host KO mouse significantly increased new bone formation and led to marked recovery of periosteal bone formation on day 21 (Figures 6A, B). Histological analysis further revealed marked and localized induction of MCP-1 expression in the callus and endosteum around the WT-derived graft in the host MCP-1^−/−^ mouse (Figure 6C). By contrast, no MCP-1 expression was observed in the callus and endosteum of the host bone in the same section.

### Reduction in periosteal bone formation in grafts from MCP-1-deficient mice implanted into WT mice: loss-of-function

To confirm whether MCP-1 is a crucial chemokine in the fracture healing process, we performed loss-of-function studies using WT-to-WT and KO-to-WT bone graft models. Transplantation of a WT donor graft into a WT host mouse led to abundant new bone formation and a bridging callus around the WT-derived graft on day 21 (Figures 6D, E). By contrast, transplantation of a KO-derived graft into a WT host markedly reduced the amount of periosteal bone formation in the donor graft.

## Discussion

Our data highlight crucial roles of the MCP-1/CCR2 axis in the early phase of fracture healing. Compared with no fracture, the expression levels of many inflammatory chemokines increased on day 3 after fracture. In particular, *MCP-1* and *MCP-3* expression were temporarily up-regulated in the early phase of fracture healing (Figure 1). Then, we found that deletion of either MCP-1 or CCR2 caused delayed fracture healing (Figure 4), and that, blockade of CCR2 only in the early phase of healing caused delayed fracture healing (Figure 5). Taken together, these results suggest that the temporary increase of MCP-1, MCP-3 and CCR2 expression in the early inflammatory phase may play a pivotal role for successful fracture healing. A recent study reported that deletion of CCR2 induces delayed fracture healing because of a decreased ability to resorb bone by osteoclasts in the remodeling phase [28]. The persistent cartilage fracture healing phenotype could be caused by defects in chondroclast/osteoclast chemotaxis that delays vascular invasion, calcification and/or remodeling. However, MCP-1 expression is known to induce the early inflammatory phase [29], when osteoclasts do not play major roles in fracture healing. Consistent with the findings of previous studies, our data show that *MCP-1* and *MCP-3* mRNA were up-regulated on day 3 (Figure 1) and that localized MCP-1 and MCP-3 expression were increased in the periosteum and endosteum in the early phase of fracture healing (Figure 2A). This suggests that increased MCP-1 and MCP-3 expression in the early inflammatory phase may be essential for normal fracture healing.

MCP-1 and its receptor CCR2 are involved in recruitment of various cells, including leukocytes, BMSCs and hematopoietic stem cells [30]–[33], and in the regeneration of damaged tissues [34], [35]. As established in earlier developmental studies, CCR2 is necessary for organ-specific homing of bone marrow-derived pluripotent mesenchymal stem cells into damaged tissues [36], [37]. Consistent with this finding, our data showed that *CCR2* mRNA was induced in the mBMSCs derived from WT mice (Figure 3B). We also found that MCP-1, but not MCP-3, induced the migration of mBMSCs in a dose-dependent manner and that *in vitro* migration was markedly inhibited by a CCR2 antagonist (Figure 3A). Therefore, this axis may be a potent candidate in the development of stem/progenitor cell-based therapy for improving fracture healing.

We have previously demonstrated that SDF-1 is induced in the periosteum during bone injury and promotes endochondral bone repair by recruiting mesenchymal stem/progenitor cells to the site of injury. In the PCR array, an increased level of SDF-1 was not observed during fracture healing in this current study, especially in the early inflammatory phase. This inconsistency may be explained partly by the differences between the presence of the unimpaired bone marrow in simple fracture healing and bone graft healing with an intramedullary nail. Moreover, the previous study investigated allograft healing, in which the surgical site is greatly avascular, and under hypoxia this induces hypoxia-inducible factor-1 activation and subsequent SDF-1 up-regulation. Hence, we consider that the MCP-1/CCR2 axis is a crucial signaling pathway during the normal fracture healing process.

Previous studies have demonstrated that damage to the periosteum and bone marrow leads to impaired osteogenesis and chondrogenesis, and delays bone healing [38], [39]. Thus, the periosteum and bone marrow seem to be important sources for recruiting mesenchymal stem/progenitor cells or osteogenic progenitor cells for promoting osteogenesis and chondrogenesis. In this study, we found that the expression of CCR2 increased transiently in the bone marrow in the early inflammatory phase and that the expression of MCP-1 also increased transiently in the periosteum and endosteum during the same period (Figure 2). We also found that WT mouse-derived bone graft markedly increased new bone formation and promoted successful fracture healing, whereas the MCP-1^−/−^ mouse-derived bone graft caused less new bone formation and delayed fracture healing (Figure 6). Importantly, although other osteogenic factors were present at the fracture site, they could not compensate for the lack of MCP-1. Collectively, these findings indicate clearly that increased MCP-1 expression in the periosteum and endosteum recruits CCR2-expressing cells and is essential for successful fracture healing.

This study has several limitations. First, we did not fully analyze the functions of other ligands for CCR2, such as MCP-3 and MCP-5, which may have roles different from those of MCP-1 in fracture healing. However, CCR2 KO mice showed similar impairment of bone healing compared with MCP-1^−/−^ mice. Therefore, it is reasonable to consider that other ligands may also have similar functions. Second, the MCP-1/CCR2 axis may have a function other than the recruitment of progenitor cells in the early phase of fracture healing, such as promoting angiogenesis. Several studies report the role of the MCP-1/CCR2 axis in angiogenesis, but not in fracture healing. This point should be clarified in the future. Lastly, we did not elucidate the cell source(s) of mesenchymal stem/progenitor cells for fracture healing in this study. Recent reports, including ours, indicate the periosteum is the key source of potent cells [19], [40], but this requires further investigation.

In conclusion, we have shown that increased expression of MCP-1 in the early phase plays a pivotal role in fracture healing by recruiting CCR2-expressing cells derived from surrounding tissues. The MCP-1/CCR2 axis is a potential target for achieving successful fracture healing. Further studies are needed to understand the functional relevance of the MCP-1/CCR2 axis in fracture healing.

## Supporting Information

Figure S1 **Immunohistochemical analysis of WT unfractured rib.** **A, B, C,** Low expression levels of MCP-1, MCP-3 and CCR2 were observed at the periosteum in the unfractured rib.
(TIFF)

Figure S2 **Effects of MCP-1 on osteogenesis, and chondrogenesis.** **A**: mBMSCs were cultured in osteoinduction media with or without MCP-1 for 14 days and stained with alizarin red S. The expression of each gene was analyzed by quantitative RT-PCR. (*n* = 5, respectively). **B**: ATDC5 cells were induced chondrocyte differentiation. MCP-1 (0, 20, 100 or 200 ng/ml) was simultaneously added every 2 days with the medium change. On days 28 after plating, cells were harvested, and the expression of each gene was analyzed by quantitative RT-PCR. (*n* = 6, respectively).
(TIFF)

Figure S3 **A, C: Histology of the fracture callus stained by hematoxylin-eosin/alcian-blue staining on day 7 (A) or day 21(C).** **B**: Histology of the fracture in MCP-1 or CCR2 KO stained by hematoxylin-eosin on day 25 (left panel) or 23 (right panel).
(TIFF)
