# Supplementary figures and images for: MCP/CCR2 Signaling Is Essential for Recruitment of Mesenchymal Progenitor Cells during the Early Phase of Fracture Healing
Source: PLoS One. 2014 Aug 18;9(8):e104954. doi: 10.1371/journal.pone.0104954 (PMC4136826; doi:10.1371/journal.pone.0104954)

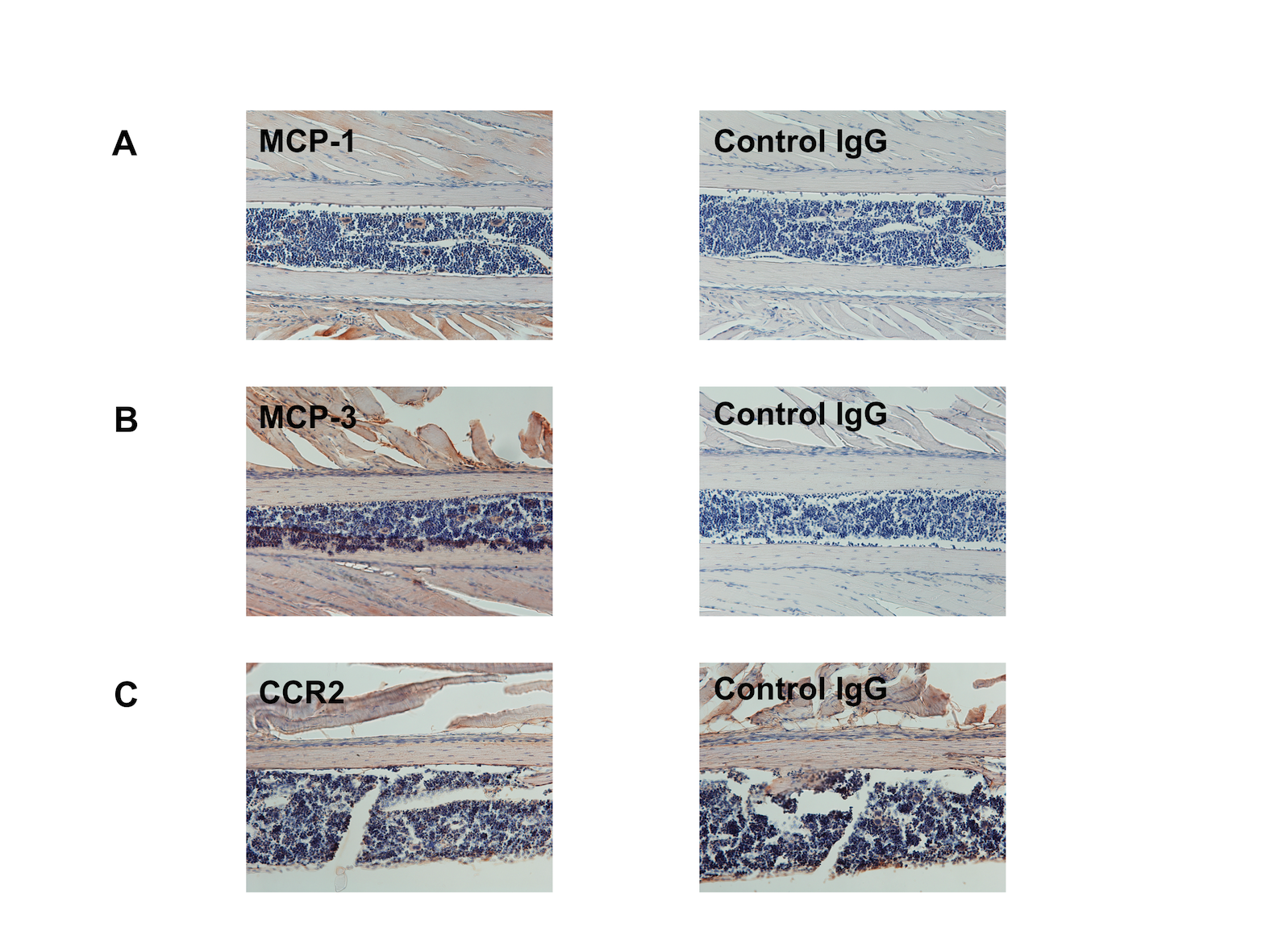

Supplement: Figure S1 — Immunohistochemical analysis of WT unfractured rib. A, B, C, Low expression levels of MCP-1, MCP-3 and CCR2 were observed at the periosteum in the unfractured rib. (TIFF) [file pone.0104954.s001.tiff]

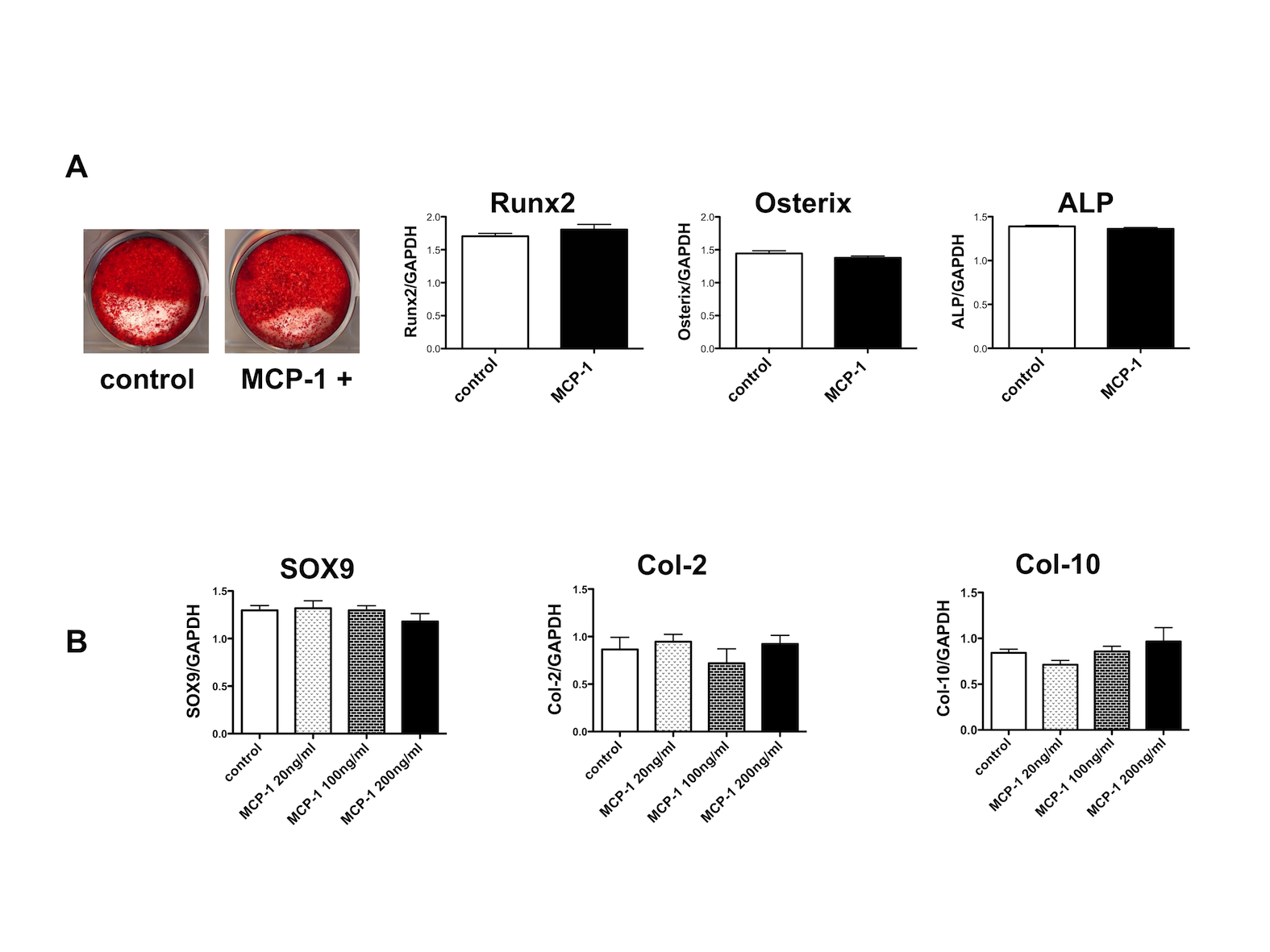

Supplement: Figure S2 — Effects of MCP-1 on osteogenesis, and chondrogenesis. A: mBMSCs were cultured in osteoinduction media with or without MCP-1 for 14 days and stained with alizarin red S. The expression of each gene was analyzed by quantitative RT-PCR. (n = 5, respectively). B: ATDC5 cells were induced chondrocyte differentiation. MCP-1 (0, 20, 100 or 200 ng/ml) was simultaneously added every 2 days with the medium change. On days 28 after plating, cells were harvested, and the expression of each gene was analyzed by quantitative RT-PCR. (n = 6, respectively). (TIFF) [file pone.0104954.s002.tiff]

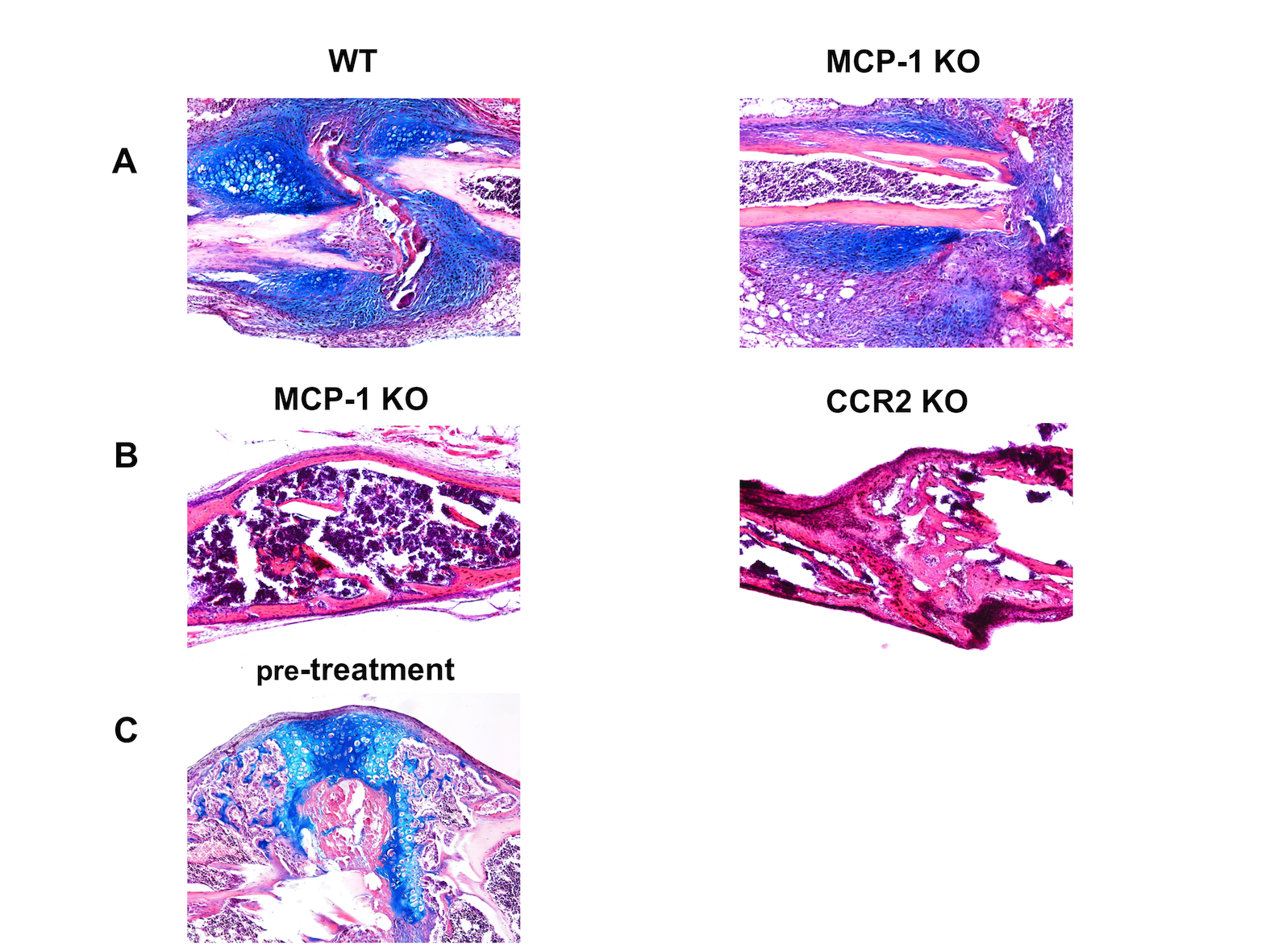

Supplement: Figure S3 — A, C: Histology of the fracture callus stained by hematoxylin-eosin/alcian-blue staining on day 7 (A) or day 21(C). B: Histology of the fracture in MCP-1 or CCR2 KO stained by hematoxylin-eosin on day 25 (left panel) or 23 (right panel). (TIFF) [file pone.0104954.s003.tiff]
